# A Bird’s Eye View: A Close Look into Avian CAM Models for Translational Blood Cancer Research

**DOI:** 10.3390/cancers18020209

**Published:** 2026-01-09

**Authors:** Izabela M. Cymer, Niamh McAuley, Cathy E. Richards, Hanne Jahns, Siobhan V. Glavey, Ann M. Hopkins

**Affiliations:** 1 Department of Surgery, RCSI University of Medicine and Health Sciences, D09 YD60 Dublin, Ireland; izabeladrozdz@rcsi.ie (I.M.C.); niamhmcauley22@rcsi.ie (N.M.); 2 Department of Pathology, RCSI University of Medicine and Health Sciences, D09 YD60 Dublin, Ireland; siobhanglavey@rcsi.ie; 3 Beaumont RCSI Cancer Centre, D09 V2N0 Dublin, Ireland; 4 School of Dentistry, RCSI University of Medicine and Health Sciences, D02 YN77 Dublin, Ireland; catherinerichards@rcsi.ie; 5 Pathobiology Section, UCD School of Veterinary Medicine, University College Dublin, D04 V1W8 Dublin, Ireland; hanne.jahns@ucd.ie

**Keywords:** haematology, chick embryo chorioallantoic membrane, CAM model, leukaemia, lymphoma, multiple myeloma

## Abstract

The chorioallantoic membrane (CAM) is a well-vascularised extra-embryonic membrane that supports avian embryonic development and can be used as an implantation site for xenograft models of various cancers. CAM tumour research models are powerful and versatile, offering a rapid, cost-effective and ethical complement to mouse xenograft studies. Their capacity for real-time observation of tumour growth, angiogenesis and metastasis within an immunocompetent living organism is particularly compelling. While CAM models have been extensively utilised for investigating solid cancers, such as breast, lung and pancreatic, their potential for haematological malignancy research remains comparatively underexplored. This review examines the relevance, advantages and translational potential of avian CAM models in studying blood cancers. Their applications across three primary categories are discussed—leukaemias, lymphomas and myelomas—highlighting experimental approaches that replicate aspects of human disease progression and therapeutic responsiveness. Moreover, the review evaluates species-specific considerations relevant to model fidelity, including evolutionary distance and functional parallels between avian and human haematopoiesis. These comparisons underscore both the opportunities and limitations for utilising CAM models in haematologic malignancy research. For their potential to investigate mechanisms of cancer development and treatment in simple but immunocompetent in vivo settings, we propose that CAM tumour models offer high value as a bridge between in vitro and mammalian in vivo studies for haematology translational research.

## Simple Summary

The incidence and mortality associated with haematological malignancies are significant, and there is a pressing need for fast, scalable in vivo experimental models that overcome some of the drawbacks of existing mouse models. This review explores the utility of the chorioallantoic membrane (CAM) in immunocompetent avian embryos as an alternative site for the growth of blood cancer xenografts. Despite being under-utilised for research in blood cancers relative to carcinomas, CAM models show much promise in recapitulating key elements of disease biology and treatment responsiveness in a simpler in vivo system, which falls outside the jurisdiction of traditional animal research parameters. This review summarises the evidence of their successful use for leukaemia, lymphoma, and multiple myeloma research to date, and offers perspectives on their limitations and necessary considerations for future translational research involving CAM models.

## 1. Introduction

Blood cancers, broadly categorised into leukaemias, lymphomas, and myelomas, represent a diverse group of malignancies originating from haematopoietic and lymphatic cells [1,2]. Over 120 different types of blood cancers have been described [3], with leukaemia, non-Hodgkin’s lymphoma, Hodgkin’s lymphoma and multiple myeloma collectively accounting for >1.2 million incidences and 700,000 deaths worldwide in 2020 alone [4]. This represented approximately 10% of all cancers recorded that year [4], with similar incidence rates recorded for the US [5,6] and the EU in 2022 [7]. The incidence and mortality rates for blood cancers are higher in men than in women [4].

Most blood cancers begin where all blood cells are made: the bone marrow. In a healthy person, white blood cells are continuously produced to replace those that are no longer viable or functional. However, leukaemia patients experience the overproduction of white blood cells from either the myeloid or lymphoid lineages, lymphoma involves abnormalities in lymphocytes, while myeloma arises from abnormal plasma cell proliferation [8,9] (Table 1). Neoplastic proliferation highlights two key hallmarks of cancer, namely sustaining proliferative signalling and resisting cell death, as neoplastic cells bypass regulatory checkpoints and evade apoptosis [10,11]. The systemic nature of blood cancers poses a significant challenge in understanding their pathophysiology and developing effective treatment strategies [12]. Unlike solid tumours, which often induce angiogenesis to sustain growth within a localised area [10,13], the dispersal of blood cancer through haematopoietic and lymphatic cells makes it difficult to follow disease progression and to isolate specific microenvironmental causative factors [14]. Treatment avenues are also complicated by widespread disease progression, affecting multiple organs such as the spleen, liver and lymph nodes [14]. Moreover, blood cancers often evade immune destruction either by downregulating antigen presentation mechanisms, by directly manipulating T- or NK-cell activity, or by disrupting immune surveillance mechanisms through the PD-1/PD-L1 pathways [10,15]. This, in turn, can influence the surrounding microenvironment, inducing tumour-promoting pro-inflammatory events [16].

Furthermore, blood cancers often present significant heterogeneity in cell populations. Distinct clonal expansions underscore the diversity, complexity, and dynamic nature of individual blood cancers [17], further complicating the study of cancer progression and the prediction of responsiveness to therapy, while highlighting the unlikelihood of universal therapeutic targets [18,19,20].

Although there are numerous factors that influence a patient’s overall survival, including patient-related factors like age or comorbidities, disease-related factors like disease burden or cytogenetic risk, and treatment-related factors such as response or resistance, the prognosis remains poor for patients diagnosed with certain blood cancers (Table 1).

**Table 1:** Overview of common blood cancers: classification, diagnosis, treatment, and survival.

	Cancer Type	Main Cell Type Affected	Symptoms	Diagnostic Methods	Median Age at Diagnosis	Common Treatment Regime	5-Year Survival Rate
Leukaemia	Acute Lymphocytic Leukaemia (ALL)	Immature B lymphocytes (lymphoblasts)	Fatigue, frequent infection, bruising, bone pain, swollen lymph nodes	Blood tests (CBC), BM biopsy, cytogenetic analysis	Younger patients, (17 years) [21]	Antimetabolites/Chemotherapy, Steroids, Asparaginase-Specific Enzyme Therapies, Targeted therapy (CAR-T, Bispecifics), Anthracyclines, SCT	~90% (children), ~60% young adolescent, ~20–40% (adults) [21]
	Chronic Lymphocytic Leukaemia (CLL)	Mature B lymphocytes	Fatigue, swollen lymph nodes, night sweats, weight loss, frequent infections	Blood tests (lymphocytosis), BM biopsy, biomarker testing	Older adults (~70 years) [22]	Targeted therapy (Bruton Tyrosine/Phosphatidylinositol 3- Kinase/B-cell lymphoma 2- Inhibitors). Monoclonal Abs, Chemo(immuno-)therapy	~90% [23], (advanced/cytogenetic dependent reduced survival) [22,24]
	Acute Myeloid Leukaemia (AML)	Myeloid precursors (myeloblasts)	Fatigue, fever, infections, anaemia, easy bruising, bleeding	Blood tests, BM biopsy, cytogenetic/biomarker analysis	Older adults (~69 years) [25]	Induction chemo (cytarabine + anthracycline) (7 + 3 regimen), targeted therapies (e.g., FLT3 inhibitors), SCT	~33% [25]
	Chronic Myeloid Leukaemia (CML)	Myeloid cells (granulocytes)	Fatigue, weakness, bone pain, enlarged spleen, unexplained weight-loss	Blood tests, BM biopsy, cytogenetic/biomarker analysis	Older patients (~66 years) [26]	Targeted therapy (tyrosine kinase inhibitors), Immunotherapies, ASCT	~71% [26]
Lymphoma	Hodgkin’s Lymphoma	Mature B lymphocytes (Reed-Sternberg cells present)	Rapidly enlarging lymph nodes, night sweats, fever, weight loss, persistent cough	Biopsy of affected lymph node, PET/CT scan, biomarker analysis	Younger adults (~39 years) [27]	Chemotherapy, radiation, immunotherapy, SCT	~90% [27]
	Non-Hodgkin’s Lymphoma	Mature B lymphocytes (Reed-Sternberg cells absent)	Painless swollen lymph nodes, fatigue, fever, night sweats, cough, abdominal pain	Lymph node biopsy, biomarker analysis, imaging	Middle-aged to older adults (~68 years) [28]	Chemoimmunotherapy (e.g., R-CHOP), radiation, SCT	~74% [28]
Myeloma	Multiple Myeloma (Plasma Cell Myeloma)	Plasma cells	Bone pain, fractures, fatigue, anaemia, kidney dysfunction (CRAB symptoms)	Blood tests (M-protein, free light chain), BM biopsy, skeletal survey	Older adults (median ~70 years) [29]	Proteasome inhibitors, immunomodulators, Steroids, monoclonal Ab therapy, ASCT	~62% [29]

To study the mechanisms underlying these complex hallmarks and to develop effective therapies, current laboratory models for haematological research will be discussed next.

## 2. Laboratory Models for Research in Haematological Cancers

### 2.1. Mouse Models for Haematological Research

In vivo models have been instrumental in advancing our knowledge of haematological malignancies, particularly humanised rodent models, which allow for detailed study of disease progression, metastasis, and therapy responsiveness [30,31,32].

Some key advantages of rodent blood cancer models include their genetic tractability and physiological similarity to humans [32,33]. These features enable researchers to replicate human disease conditions, including genetic alterations and tumour microenvironments, thus serving as a foundation for preclinical drug development. However, rodent blood cancer models also have some significant limitations, including the following:

*Cost:* Rodent models are expensive to establish and maintain, particularly for cancers with long latency periods [34] or when specific pathogen-free (SPF) facilities are required to house transgenic and immunodeficient strains used in haematological malignancy research [34,35,36]. High costs impact scalability, potentially restricting comprehensive drug testing and mechanistic studies [35,37].

*Model incompatibility:* Some immunodeficient mouse models, such as Foxn1-/- nude and C.B17-SCID mice, are unsuitable for haematological malignancy research due to their limited immune cell interaction with tumour cells. Foxn1-/- nude mice lack a thymus and T-cells, but retain residual immune responses, restricting tumour cell engraftment. C.B17-SCID mice can develop functional T- and B-cells with age, complicating long-term blood cancer studies via cytotoxicity to transplanted cells [34,38]. Furthermore, as the immunotherapy era continues to revolutionise blood cancer treatment, studying immunotherapeutic treatments in immunodeficient mouse models, despite their suitability for patient-derived xenografting (PDX), remains challenging. Of course, mouse models are plastic and may be humanised to form a representative immune system, but the creation of such resources is complex, timely and costly [39].

*Genetic incompatibility:* Inbred murine models lack genetic diversity, which limits generalisability in capturing disease mechanisms or drug responsiveness at the cancer patient population level [40].

*Ethical, cost, and time considerations:* In Europe, Directive 2010/63/EU ensures the protection and care of animals used in research. This directive enforces the 3R principles (Replacement, Reduction and Refinement) and mandates ethics committee reviews, licencing, and inspections for facilities conducting animal experimentation.

Of note, in 2016, the Dutch government expressed intention to make the Netherlands “largely free of animal testing” by 2025 [41]. However, achieving this goal has proven challenging. In fact, over 400,000 animal tests were conducted in the Netherlands in 2022 alone [42,43], with some EU safety regulations mandating animal testing for new chemical substances, hampering the Netherlands’ ability to unilaterally eliminate such practices [44]. While the nation has looked into alternatives, their validation is in its infancy and approval processes can take decades. Accordingly, the goal of freedom from animal testing has been delayed.

The ease of genetic manipulation in mice has long been considered a key advantage of murine systems in recapitulating human disease and one that alternative vertebrate models lack the capacity to compete with. However, this technical gap is closing, thanks to recent advances in primordial germ cell genome engineering that have made genetic manipulation increasingly feasible in chickens. For example, efficient germline transgenesis has been demonstrated using lentiviral vectors in chickens, with good conservation profiles (and no detectable silencing of transgene expression between generations) proving particularly promising [45]. Moreover, McGrew et al. showed that expression of both *lacZ* and GFP in embryos and birds mirrored the expression patterns of those in transgenic mice [45]. Transgenic birds produced at the UK National Avian Research Facility (The Roslin Institute, University of Edinburgh) have led the field for their capabilities to directly track host lineages that contribute to tumour engraftment and vascular remodelling in vivo. An early success was the Roslin Green cytoplasmic GFP chicken line, a ubiquitous reporter expressed in extra-embryonic tissues that was used for fate mapping and grafting paradigms [46,47]. The use of Roslin Green chicken embryos in CAM assays allows for unambiguous discrimination between host stromal/vasculature elements and implanted tumour material, which is a step change in this field. Cell lineage-restricted immune reporters have also been generated, including the CSF1R-macrophage reporter line [47,48,49,50], which provides in vivo visualisation of mononuclear phagocytes that may be repurposed to measure myeloid recruitment, perivascular localization, or tumour-associated macrophage dynamics during CAM tumour growth and angiogenesis assays.

Collectively, this highlights the value of complementary and alternative models that can bridge the gap between in vitro studies and in vivo validation or even between in vitro studies and pre-clinical in vivo validation in rodents. For a more complete summary of rodent models in blood cancers, the reader is directed to several excellent reviews [51,52,53,54,55]. Instead, the current review will evaluate the potential of the chick embryo chorioallantoic membrane (CAM) model to bridge the gap between in vitro and in vivo validation studies for blood cancer research, in line with the 3Rs principles.

### 2.2. The CAM Model as a Gateway to Living Systems

Chick embryo development is often described in Hamburger–Hamilton stages, a standardised developmental staging system based on morphological criteria rather than chronological age. For a full description of Hamburger–Hamilton staging, interested readers are directed to two excellent articles [56,57]. While species-specific staging resources are available for quail, turkey and ostrich, their coverage and granularity differ by species. Developmental stages are best defined for quail [58,59], turkey [60,61] and ostrich [62,63,64], but published quail, turkey and ostrich research resources in the CAM field are often anchored to “day of incubation” and morphometric data. Accordingly, for reasons of both practical implementation and awareness of EU scientific animal protection legislation, this review principally discusses CAM development in “embryonic development days”. The chorioallantoic membrane (CAM) of chick development forms following the fusion of two extraembryonic tissues, the allantois and chorion, and appears at embryonic development day (EDD) 3.5 [65,66,67,68,69]. The CAM contains all three germ layer cell types: the allantoic endoderm, allantoic and chorionic mesoderms, and chorionic ectoderm [70]. This double-layered membrane connects directly to the allantoic arteries and vein, making it a highly vascularised structure that functions like a homologue of the human placenta in protecting and nourishing the developing embryo [67,69]. An exponential growth phase occurs in the CAM between EDD3 and 10, culminating in its full differentiation by EDD13 [65].

Notably, chick embryos have been used for decades to generate preliminary insights into the toxicity of drugs or exogenous chemicals, offering a valuable platform for preclinical toxicology studies.

However, in 1911, the chick embryo CAM was first used by Rous and Murphy as a tumour xenograft site, supporting the growth of chick sarcoma tumours [71,72]. CAM tumour models have since been extensively used for the study of the core hallmarks of cancer, across various solid and liquid cancers, and extensively reviewed by recent experts [39].

With this in mind, the CAM model provides a promising complement/alternative as a cost-effective and scalable in vivo model for studying blood cancers [73,74,75,76,77,78].

Some of its advantages are as follows:

*Cost:* Chick embryos are cheap, easily purchased, and require no specialised husbandry.

*Fast, easy, and accurate data acquisition:* The short gestational timeline of the chicken embryo (21 days) allows for faster experimental turnover compared to rodents.

*Dynamic Observation:* The highly vascularized CAM supports tumour grafting, and its accessibility through a “window” cut into the eggshell permits real-time visualisation of tumour growth and angiogenesis. Furthermore, CAM-grown tumours have been noted to histologically and morphologically resemble those at primary tumour sites [79], and metastasis to end organs can be readily studied. For video representations of the experimental methodologies involved, the reader is directed to an excellent publication in the Journal of Visualised Experimentation that demonstrates the practicalities involved [40].

*Transient immunodeficiency at implantation*: Avian embryos are highly tolerant to non-host cancer cell implantation on the CAM early during development [39,69]. This is because avian T-cells principally develop functionality in the third and final trimester of development [80]. That avian embryos possess both innate and adaptive immune systems is also unknown to many. In fact, the term “B-cell” derives not from the human bone marrow location where these cells mature, but rather from the avian lymphoid organ known as the “Bursa of Fabricius”, where B cells were first identified [81,82].

*Genetic Background:* Most embryos utilised in CAM experiments are sourced commercially and are not inbred, offering genetic variability that better recapitulates biological diversity and responsiveness to treatment (including tumour evolution). Additionally, since embryos develop outside of the maternal environment, minimising the influence of sex hormones that affect differentiation later in development, the model reduces gender bias and provides a mixed population of responses to therapies [83]. However, gender manipulation and phenotypical sex reversal are theoretically still possible in chicken embryos, further complementing model plasticity [84,85,86].

*Ethical Considerations:* In most jurisdictions, CAM experiments can be undertaken without the need for scientific animal protection licences as long as the chick is not allowed to hatch. This not only speeds up workflows but is compliant with the 3R principles of Reduction, Replacement, and Refinement [39].

*Toxicity Assessment:* As a fully integrated and vascularised system, the chick enables the evaluation of compound toxicity in a living biological system prior to progression into vertebrate models. This makes it particularly valuable for early-stage dose-finding and safety screening. The ability to observe local and systemic responses in real time—including tissue damage, haemorrhage, inflammation, and lethality—allows for the determination of tolerable dose ranges with a high degree of translational relevance. Importantly, this approach reduces the number of higher-order animals required for preliminary toxicological studies.

### 2.3. Histopathological Concordance Between Murine and CAM Xenografts

Direct structural/functional comparisons between haematological malignancies engrafted in CAM versus murine xenograft model systems have not yet been reported. Their lack is an impediment to an evidence-based decision framework for the utilisation of CAM models in blood cancer research, which would build on decades of research with well-characterised murine models. Encouragingly, however, there is some work demonstrating that solid tumour xenografts generated from cell lines or patients exhibit a high degree of histopathological concordance when grown on the CAM versus in mice. Across these investigations, tumour architecture, cytological features, proliferative indices and tumour–stroma organisation have been reported to be largely conserved between avian and murine hosts. These studies collectively establish a precedent for cross-platform morphological equivalence and provide a compelling rationale for extending such comparative analyses to blood cancers as CAM models continue to mature in this space.

Specifically, Ranjan et al. [87] compared parallel xenografts of human breast cancer cell lines representing distinct molecular subtypes grown on the CAM and in immunodeficient mice. Xenograft tumours generated in both systems largely retained comparable levels of differentiation, tumour grade and cellular density. In addition, the histomorphology of MDA-MDA-231 CAM xenografts was similar to triple-negative breast cancer patient tumour tissue in terms of high pleomorphism and cord-like tumour cell arrangements surrounded by fibromyxoid stroma. Furthermore, diagnostic and prognostic biomarkers such as oestrogen/progesterone receptors and the Ki67 proliferation index were conserved between the two models, leading the authors to conclude that mouse CAM tumours accurately represented the major morphological features seen in murine models and their corresponding human tumours [87]. Similarly, Pinto et al. [79] reported similarities in tumour morphology and stem cell marker patterns between CAM- and mouse-grown xenografts of organotropic breast metastatic cell populations [79]. Encouragingly, similar implantation loads (1 × 10^6^ cells) yielded comparable xenografts even when grown for different time periods (CAM: 7 days vs. mouse: 3 weeks). Thus, the study concluded that CAM xenografts could reproducibly model many aspects of the complex phenotypes of stem-like cancer cell populations seen in murine models [79]. Finally, metastatic clear cell renal cell carcinoma (ccRCC) research conducted by Ishihara et al. [88] further supports the generalisability of CAM versus mouse-grown tumour xenograft research. Specifically, CAM tumour xenografts grown over 10 days phenocopied the growth and metastatic behaviour of genetically modified RENCA cells grown in SCID mice over 3–6 weeks and at a fraction of the cost (estimated as $100 per mouse versus $1 per egg) [88].

Collectively, these studies demonstrating histopathological concordance between multiple CAM- versus mouse-grown solid tumours support the evaluation of CAM models to complement mouse xenograft models for haematopoietic malignancy research. This also aligns with best-practice scientific and ethical principles of reducing animal usage and increasing experimental efficiency and scalability.

### 2.4. Evolutionary Distance and Functional Relevance (Or Model Relevance): Avians and Humans Compared

The avian immune system and its associated lymphomyeloid organs are situated phylogenetically between those of reptiles and humans. Similarly to humans, the avian adaptive immune system relies on two main cell types, T-cells and B-cells. Primary (central) lymphoid organs are the thymus, bone marrow, and the Bursa of Fabricius. The latter is a feature unique to birds, responsible for the amplification and differentiation of B-cell progenitors. In humans, this process occurs in the bone marrow. The yolk sac is the primary haematopoietic site in the chicken embryo between EDD4 and EDD12 [89,90]. Haematopoiesis then occurs in the bone marrow and yolk sac between EDD13 and EDD20 [90]. Further, the spleen has an important role in embryonic lymphopoiesis, as B-cell progenitors undergo rearrangement of their Ig genes before colonising the Bursa of Fabricius [91].

Secondary (peripheral) lymphoid organs are widespread in birds and consist of the spleen, caecal tonsils, Peyer’s patches and lymphoid aggregates in the glands and parenchymal organs. These are populated by T- and B-cells after their development in the thymus and Bursa of Fabricius, respectively. Well-developed lymph nodes are absent in most avian species, including the chicken, giving the spleen a dominant role in the generation of immune responses [92].

Many of the cytokines and chemokines that act as extracellular signals in immunological development and in immune responses identified in mammals are also present in the chicken, although these have only 25–35% amino acid identity with their mammalian orthologs [93].

In galliformes, naturally occurring blood cancers are common and are mainly virus-associated. The three main neoplasias are as follows: (1) Marek’s disease-associated T-cell lymphomas, (2) avian leukosis tumours of B-cells and other haematopoietic cells, and (3) reticuloendotheliosis virus-induced tumours characterised by a variety of syndromes, including lymphoid neoplasia [94]. Similar disseminated distribution of malignant immune cells affecting lymphoid and other parenchymal organs, including skin and nerves, has been described for all three neoplastic diseases [95].

Cytokines play a crucial role in the growth, survival, and dissemination of malignant plasma cells in human patients of multiple myeloma (MM); for example, Vascular Endothelial Growth Factor (VEGF), Interleukin-6 (IL-6), Tumour Necrosis Factor-alpha (TNF- α), B-cell activating factor (BAFF) and Receptor Activator of Nuclear Factor-κB ligand (RANKL) have all been identified in promoting myeloma intravasation [96,97]. Furthermore, the CXCR4/CXCL12 axis plays a pivotal role in proliferation, invasion, dissemination and drug resistance in human MM patients [98]. Interestingly, VEGF and NF-κB signalling pathways have also been identified as pathways involved in cellular transformation by a Marek’s Disease Virus Oncoprotein in chicken [99]. Further, IL-6, TNF-α, RANKL, BAFF receptors, and CXCR4 have been found to be present in the chicken genome [93].

### 2.5. Species-Specific Considerations in CAM Models of Haematologic Malignancy

Whether rodent or avian, careful selection of the most appropriate model for cancer xenograft studies is of pivotal importance. Avian CAM models have been developed not only in chickens, but also in quail, duck, turkey, emu and ostrich species. These models not only differ in egg size but also in the duration of embryonic development, which, in turn, dictates the feasible length of an experiment. For example, chick embryos hatch at ~21 days, with most experimental work conducted up to 18 days. Quail embryos typically hatch after ~18 days (sometimes as early as 16.5), with experimentation usually concluding by EDD 11. Duck and turkey embryos require ~26–28 days of incubation, with experimentation ending around EDD 24. In contrast, ostrich embryos hatch after ~42–46 days, while emus require ~56 days. Such variations offer opportunities in tailoring individual CAM models for specific research applications—for example, long-term studies involving radiation or chemotherapy may benefit from longer-incubation models. Conversely, toxicity assays and anti-angiogenic studies can be efficiently tested in shorter-incubation models. For studies of haematological malignancies, intermediate-incubation models, such as chick and turkey embryos, appear particularly well suited. For a comprehensive review on chick, quail, turkey and egg embryonic/CAM development, as well as their uses, please refer to references [100,101,102].

The chicken CAM model is the standard and most well-characterised platform for in ovo tumour studies. One review has stated that ~95% of CAM studies use chick embryos, and only ~4% use quail—although turkey, duck or emu models were not captured [102]. Nonetheless, it is evident that other avian models are understudied across all cancers, let alone blood malignancies. From a technical standpoint, this is reasonable, as chicken eggs are robust and easier to procure than turkey, duck, ostrich or emu eggs. Importantly, the chick embryo also remains largely immunologically tolerant until late in gestation, permitting xenograft growth for approximately one week without immunological rejection. This window enables rapid tumour vascularisation and expansion prior to the development of functional immunocompetence; consequently, chicken CAM models can recapitulate features of angiogenesis, invasion, and metastasis that are difficult to achieve in vitro. Notably, up to 80% of tumour cells injected intravenously (e.g., into the allantoic vein) survive in the circulation and extravasate within 1–3 days—a far higher efficiency of dissemination than in adult mouse models [101]. In summary, the ease of use, low cost, rapid tumour formation, and ability to model metastasis in the chicken CAM model make it a highly feasible platform for translational haematologic cancer research.

Quail embryos account for only a minority of CAM studies, despite offering certain practical advantages. The quail eggshell is thinner and easier to open, which simplifies experimental access to the CAM and can improve the success of shell-less ex ovo culture [100]. In fact, quail embryos are small enough to fit into 6-well tissue culture plates, saving incubator space and enabling higher-throughput drug testing [103]. The fundamental biology of the quail CAM is similar to that of the chick, and it likewise tolerates xenograft engraftment. Tumours grafted onto the quail CAM develop faster in absolute terms, and indeed quail embryos enter later developmental stages a few days before chickens. However, while this accelerated timeline can be an asset for rapid assays, it also means shorter experimental windows post-grafting. Nonetheless, quail CAM models are feasible, but their use in haematologic malignancy research has been limited so far. Some studies have demonstrated successful tumour cell xenografting onto the quail CAM for angiogenesis and invasion assays [104], suggesting that, in principle, quail embryos could host blood cancer tumour cells similarly. However, this has yet to be reported. In practice, most researchers default to chickens due to familiarity and scale, but quail remains a viable alternative depending upon experiment size and timeframe. Additionally, the Japanese quail genome was sequenced in 2013 [105,106,107], facilitating molecular studies of tumour–host interactions and broadening the utility of quail CAM assays in cancer and haematologic research.

Larger bird species such as ducks and turkeys have been explored even less frequently in CAM tumour research to date; however, turkey embryos present unique attributes that have been used for a limited number of haematological malignancy studies. Owing to their proportionally larger eggs, turkey embryos have larger CAM surface areas that could, in theory, accommodate larger (or even multiple) tumour grafts, and offer better graft visualisation over longer observation windows. Additionally, the blood volume of turkey embryos exceeds that of chicks, facilitating serial sampling and simplifying intravenous cell injection or drug administration. However, the slower development of turkey embryos necessitates adjustment of the timing of interventions. For example, a comparative study found that optimal engraftment of chronic myelogenous leukaemia tumour cells occurred after injection on EDD 10 in chickens versus EDD 12 in turkeys [108]. However, engraftment was successfully detected in the turkey bone marrow as early as two days after injection, and when both chick and turkey were examined seven days after injection, engraftment levels were significantly higher in turkey embryos [108]. This positions turkey embryos as potentially high-value models for blood cancer research, although their lower availability, higher cost, and thicker shells may limit their adoption.

**Table 2:** Avian models used in human haematological research with cancer cells.

	Chick	Turkey	Quail	Ostrich/Emu
Leukaemia	✓ [109,110,111,112]	✓ [108]	✗ [113] ^†^	✗
Lymphoma	✓ [114,115,116,117]	✓ [108]	✗	✗
Myeloma	✓ [40,118,119,120,121]	✓ [108]	✗	✗

† Used to study the anti-angiogenic effect of anticancer compounds on CAM without cancer cells. ✓ indicates published data for this CAM model in the specified cancer type. ✗ indicates no published data identified.

At the time of writing this review, no articles relating to haematological malignancy research in ostrich or emu embryos were found (as summarised in Table 2). However, novel work has introduced the ostrich embryo as an innovative CAM platform for imaging applications. In ovo experiments with ostrich eggs can be performed without the need for dedicated small-animal equipment, as the ostrich egg is large enough to be imaged using standard hospital PET, CT or MRI machines [122]. In one study, methods were established for cannulating ostrich CAM blood vessels and injecting radiotracers, and whole-embryo PET/CT scans with fluorodeoxyglucose (FDG) were successfully conducted to visualise metabolic activity in vivo. Despite the obvious anatomical differences, the biodistribution of FDG in the ostrich embryo was found to be similar to that of chick embryos, rodents, and even humans, with the highest glucose uptake in the developing bone growth plates and brain [122]. Ostrich eggs offer potential to bridge a translational gap between CAM assays and imaging; for example, systemic injection of haematological cancer cells followed by whole-body PET/CT could permit observation of their homing to embryonic bone marrow or lymphoid organs and, in turn, metastasis. Notwithstanding those advantages, ostrich eggs pose their own challenges in terms of higher cost and the need for larger incubators and specialised drilling equipment to penetrate thick shells. Yet, for certain high-impact applications like performing whole-body therapeutic evaluations, the ostrich embryo offers remarkable promise. Bearing in mind that the ostrich embryo has a gestation period of ~42–46 days, it is uniquely positioned to support long-term therapeutic studies, such as a breast cancer study in which a 14-day graft period was possible [123]. The same time intervals also align better with clinical treatment cycles of patients, which last about 21–28 days. Taken together with the feasibility of ostrich CAM cannulation and repeated tracer injection/imaging [122], this suggests that longitudinal drug administration is also technically possible and a potentially attractive model for evaluating multi-cycle chemotherapy.

## 3. Modelling Blood Cancers in the CAM Model

Despite its potential, the application of the CAM model for blood cancer research remains underexplored relative to solid tumour research [102]. This gap underscores an opportunity to leverage the CAM system for gaining preliminary insights into haematological malignancies. It has been noted that the capacity of the CAM model to replicate aspects of the tumour microenvironment assists in uncovering phenomena not observable in conventional in vitro systems, thus providing a complementary bridge to more complex in vivo models [39]. These methods and applications are outlined below and summarised in Table 3.

### 3.1. Leukaemia

Leukaemia is a group of malignant disorders characterised by uncontrolled proliferation of abnormal white blood cells, disrupting normal haematopoiesis and leading to immunosuppression, anaemia and impaired blood function [124].

Although the CAM model remains underutilised for leukaemia research, a small subset of studies has nonetheless demonstrated its utility for modelling this family of haematological malignancies. Specifically, one study has demonstrated the successful engraftment of human leukaemia cell lines (K562 and DAMI) via both intra-amniotic and intravenous injections into chick embryos, as well as the demonstration of detectable tumour nodules on the CAM within seven days post-cell delivery [109]. Furthermore, the same study confirmed consistent tumour regression following treatment with the anticancer agent doxorubicin [109], highlighting the CAM model as a potential in vivo screening tool for leukaemia therapeutics (and haematological malignancy therapeutics in general).

Grinberg et al. extended this approach using turkey embryos, confirming the broader potential of avian hosts for modelling human leukaemia [108]. This study demonstrated consistent engraftment of leukaemia cell lines in both haematopoietic and extramedullary tissues of turkey embryos, plus a consistent pattern of reduced tumour load following single-dose treatment with doxorubicin [108]. The research was further supported by another study in which primary CD34+ leukaemic stem cells intravenously injected into the chick CAM evoked the detection of CD34 transcripts in embryonic haematopoietic tissues and hepatic lesions [110]. Additional evidence of tumour-host signalling was provided by a study showing that conditioned medium from Friend erythroleukaemia cells triggered angiogenesis upon application to the chick CAM [111]. Other research in this area has demonstrated anti-angiogenic therapeutic responsiveness in K562 chronic myeloid leukaemia cells implanted onto the CAM [112].

Collectively, these focused examples offer compelling evidence for embryonic avian environments as supportive platforms for leukaemic cell and stem cell colonisation, angiogenesis research, and therapeutic advancement studies.

### 3.2. Lymphoma

Lymphoma is the most prevalent blood cancer affecting lymphocytes. It can be classified into Hodgkin’s lymphoma (HL), characterised by multinucleated Reed–Sternberg (B cells) or Non-Hodgkin’s lymphoma (NHL), involving both B- and T-cells.

#### 3.2.1. Hodgkin’s Lymphoma (HL)

To elucidate the mechanistic role of macrophages in lymphoma dissemination, the CAM model has been utilised by Arlt et al. [114]. Co-inoculation of human HL L428 cells with/without CD14+ PBMCs or macrophages onto the CAM EDD 10 elicited differences in functional tumorigenic responses, with co-cultured xenografts exhibiting significant reductions in tumour size and haemorrhaging in vivo. Moreover, α-CD30 immunostaining revealed variations in tumour morphology, compartmentalization and invasion characteristics in the presence of macrophages. These in vivo findings provide a foundation for future studies on immune-mediated invasion in HL chick embryo models.

#### 3.2.2. Non-Hodgkin’s Lymphoma (NHL)

This diverse group of lymphomas is characterised by the clonal expansion of malignant T-cells in the skin. Given the high frequency of disease relapse in cutaneous T-cell lymphoma (CTCL) patients, novel therapeutic strategies are urgently needed. In one dual in vitro/in vivo study, a combination treatment with ruxolitinib and resminostat was investigated for its efficacy in inhibiting T-cell lymphoma proliferation [115]. Specifically, inoculation of MyLa or SeAx NHL cells onto the CAM on EDD 12 yielded large primary tumours weighing 100–200 mg within seven days. Topical combination treatment with ruxolitinib and resminostat evoked significant reductions in tumour AKT or ERK phosphorylation, in tumour cell extravasation from terminal CAM capillaries, and in metastasis (quantified as human-specific Alu sequences detected by PCR in downstream organs) [115]. Collectively, this underscores the value of the CAM model for assessing the therapeutic potential of JAK/HDAC inhibitor combinations (or other treatment modalities) in CTCL treatment [115].

It is noteworthy that current CTCL models mostly use severe combined immunodeficient (SCID) or athymic nude mice as tumour hosts. However, the absence of functional T-cells in these models limits their ability to accurately replicate the lymphoma microenvironment. Accordingly, the CAM model, in which functional T-cells emerge by EDD 11 and fully mature by EDD 18, offers a promising alternative as a preclinical system for establishing CTCL tumours in vivo [115].

#### 3.2.3. B-Cell Lymphoma

Burkitt lymphoma (BL) is a rare lymphoma strongly associated with Epstein–Barr Virus (EBV) infection. For most adult patients, relapse occurs within one year of remission, highlighting the need for improved understanding of BL biology. To address this, Klingenberg et al. [116] explored the suitability of the CAM as an in vivo model. Inoculation of BL2B95 cells onto the CAM yielded highly vascularized solid tumours morphologically resembling BL tumours in 100% of embryos. Moreover, serial intravital imaging between EDD 12 and 17 captured the movement of single GFP-expressing tumour cells within the CAM [116], establishing this as a viable preclinical model for studying the dissemination of individual BL cells in real time.

Following their success in establishing an in ovo model of BL, Klingenberg et al. [117] tested imipramine blue, an analogue of the tricyclic antidepressant imipramine, for its potential to impede BL dissemination to lymphatics. Imipramine blue, which has recently been investigated in medulloblastoma and CML, significantly reduced both tumour size and dissemination in the BL CAM model, underscoring the value of this model for drug screening and pharmacodynamic evaluation at clinically translatable concentrations.

### 3.3. Multiple Myeloma

Multiple myeloma (MM) is the second most common haematological malignancy, accounting for 1% of all cancers diagnosed [125]. It is characterised by abnormal proliferation of plasma B cells in the bone marrow, crowding out healthy plasma cells and other lymphocytes, as well as erythrocytes and platelets, ultimately leading to bone lesions, anaemia and persistent infections in patients [126,127]. MM can present as two precursor disease states, Monoclonal Gammopathy of Undetermined Significance (MGUS) and Smouldering Multiple Myeloma (SMM), with progression rates to active MM of, respectively, 1% and 10% per year [125]. Despite improvements in patients’ progression-free and overall survival spurred by drug treatments like Bortezomib (Velcade) and Lenalidomide (Revlimid), MM remains incurable, and nearly all patients will relapse.

#### 3.3.1. Modelling MM Angiogenesis on the CAM

Angiogenesis, the growth and remodelling of pre-existing blood vessels that facilitates tumour progression [128,129,130], has been well-studied in CAM models. In 1999, Vacca et al. reported a superior angiogenic response in the CAM vasculature following the application of conditioned medium from patients with active MM versus that from patients with non-active disease or with MGUS [119]. This further correlated with vascularisation in the bone marrow. Furthermore, Ribatti et al. showed significantly higher vasoproliferative activity between EDD 8–10 in CAM blood vessels overlain with gelatin sponges containing plasma cell suspensions from patients with active MM versus non-active MM or MGUS [120]. These data strengthened the previous findings with cell-free extracts [119,120], and, furthermore, were linked to higher mitotic activity in the CAM endothelium during this period [65]. Importantly, this work also identified a critical angiogenic window midway during development (as implants in older embryos had diminished vasoproliferative responses) and highlighted the potential role of cytokines, such as fibroblast growth factor-2 and vascular endothelial growth factor (VEGF), as drug targets to reduce MM-induced angiogenesis [120].

#### 3.3.2. MM Solid Mass Growth on CAM

Isolated solid forms of MM, known as solitary plasmacytomas, can occur in bone or soft tissue independently of haematogenous disease, but do not necessarily precede it. Nonetheless, patients with solitary plasmacytomas have an increased risk of progressing to full-blown MM, and accordingly, the CAM model has high value in recapitulating this type of tumour mass and assessing its responsiveness to treatment.

In one such study, human MM xenografts were established on the chick CAM using OPM-2 and RPMI-8226 cells pre-cultured with human mesenchymal cells in a collagen type-I matrix and implanted as spheroids atop the CAM ex ovo [40]. Using four onplants per embryo, angiogenic responses to plitidepsin and bortezomib were assessed using fluorescent stereomicroscopy. Bortezomib significantly inhibited MM xenograft growth, as measured by GFP ELISA, and Plitidepsin significantly reduced xenograft vascularisation, measured as the number of blood vessels sprouting into the onplant.

#### 3.3.3. MM Drug Testing on the CAM

Another study subsequently utilised the CAM model to test novel marine-derived compounds for inhibitory activity against MM cell-induced angiogenesis [118]. Specifically, GFP-transfected MM cell lines were combined with human bone marrow mesenchymal stromal cells into collagen matrix onplants and then grafted ex ovo onto the chick CAM on EDD 7 and examined by fluorescence microscopy and GFP ELISA after 5 days of growth. Two compounds were identified as exerting superior tumour-inhibitory effects compared to the clinically used drug bortezomib [118].

The same researchers also searched for potential anti-angiogenic activity in these compounds [118] via the “gelatin-sponge” CAM technique pioneered by Ribatti and Vacca [121,131]. Specifically, gelatin sponges soaked in a conditioned medium of bone marrow-derived endothelial cells from MM patients were grafted onto the CAM on EDD 8 and exposed to the compounds of interest. Sponges from MM patient samples induced the formation of new capillaries in a characteristic “spoked-wheel” pattern by EDD 12, which was significantly reduced after treatment with four marine compounds [118].

These results highlight the CAM model’s effectiveness in identifying compounds with both anti-tumour and anti-angiogenic properties.

#### 3.3.4. Repurposing Drugs for MM

It is an emerging trend that drugs for different blood cancers (such as the BCL-2 inhibitor Venetoclax used in leukaemia patients) are now being tested for relapsed/refractory MM patients [132,133,134]; data would suggest that the CAM model offers promise for evaluating their potential benefit. In 2019, Willenbacher et al. [135] tested pixantrone, a monotherapy approved for the treatment of adult patients with aggressive Non-Hodgkin B-cell Lymphoma, against MM onplants in the CAM model. Encouragingly, pixantrone was found to exert strong anti-proliferative activity and to significantly reduce tumour burden. This confirmed and extended parallel in vitro data for the anti-MM effects of pixantrone [135], and further supports the validity of the CAM approach as an effective in vivo model for the testing of MM disease mechanisms and drug responsiveness.

**Table 3:** Summary of CAM model conditions used for blood cancer investigations.

Cancer Type	CAM Model	Cell Line/Patient	Cell Number	Method of Delivery	EDD Inoculation	Therapy	Study	Ref.
Leukaemia	Chicken	Cell Line (K562 and DAMI)	5 × 10^5^–5 × 10^6^ K562 or DAMI	1. Cells atop CAM, 2. YS inj., 3. Amn inj., 4. IV CAM inj.,	1.EDD9–11, 2. EDD3, 3. EDD3, 4. EDD11	Chemotherapy	Intervention	[109]
Leukaemia/lymphoma/multiple myeloma	Turkey	K562, DAMI, Jurkat, HL-60, G2, CCRF, CAG, U266, Raji, HCL-2 and Primary material	1–10 × 10^6^	IV inj	EDD11–13	Chemotherapy	Intervention	[108]
Leukaemia	Chicken	MNC or CD34+ cells, Primary material	2–5 × 10^4^	IV inj	EDD11	N/A	Engraftment in organs	[110]
Leukaemia	Chicken	Friend erythroleukemia	CM	Soaked GS	EDD8	N/A	Angiogenesis	[111]
Leukaemia	Chicken	N/A	Therapeutic	Soaked GS	EDD8	d-Limonene	Angiogenesis	[112]
Lymphoma (HL)	Chicken	HL L428 cells ± CD14+ PBMCs or macrophages	2 × 10^6^	Matrigel	EDD10	N/A	Tumour growth	[114]
Cut. T-cell Lymphoma	Chicken	MyLa or SeAx	(1) 1 × 10^6^ (2) 5 × 10^4^	IV	(1) EDD10 (2) EDD12	Ruxolitinib and Resminostat	(1) tumour proliferation (2) Metastasis	[115]
Burkitt Lymphoma	Chicken	BL2B95 and BL2-GFP	10^6^	Matrigel	EDD10	N/A	Metastasis	[116]
Burkitt Lymphoma	Chicken	BL2B95	10^6^	Matrigel	EDD10	Imipramine-blue	Tumour effect + lymphogenic dissemination + angiogenesis	[117]
Multiple Myeloma	Chicken	CM from patient material	N/A	GS	EDD8	N/A	Angiogenesis	[119]
Multiple Myeloma	Chicken	Patient material	1.8 × 10^4^	GS	EDD8–12	N/A	Angiogenesis	[120]
Multiple Myeloma	Chicken	OPM-2^eGFP^ or RPMI-8226^eGFP^	2.5 × 10^5^ (MM cells) + 0.5 × 10^5^ (HMSCs)	Collagen Type I	EDD9	Plitidepsin and bortezomib	Angiogenesis, GFP expression/quantification	[40]
Multiple Myeloma	Chicken	OPM-2^eGFP^ or RPMI-8226^eGFP^ GS: CM	2.5 × 10^5^ (MM cells) + 0.5 × 10^5^ (HMECs)	Collagen Type I and GS	Collagen: EDD7, GS: EDD8	Marine compounds	Angiogenesis, GFP expression/quantification	[118]
Multiple Myeloma	Chicken	OPM-2 ^eGFP^	3 × 10^5^	Collagen Type I	EDD9	Pixatrone	GFP expression/quantification	[135]

Abbreviation s: inj: injection, YS: yolk sac, Amn: amniotic cavity, IV: intravenous, EDD: embryonic day of development, CM: conditioned medium, GS: gelatin sponge, HMSCs: human mesenchymal stromal cells, HMECs: human mesenchymal endothelial cells, N/A: not applicable.

## 4. Conclusions and Considerations

**Figure 1:**
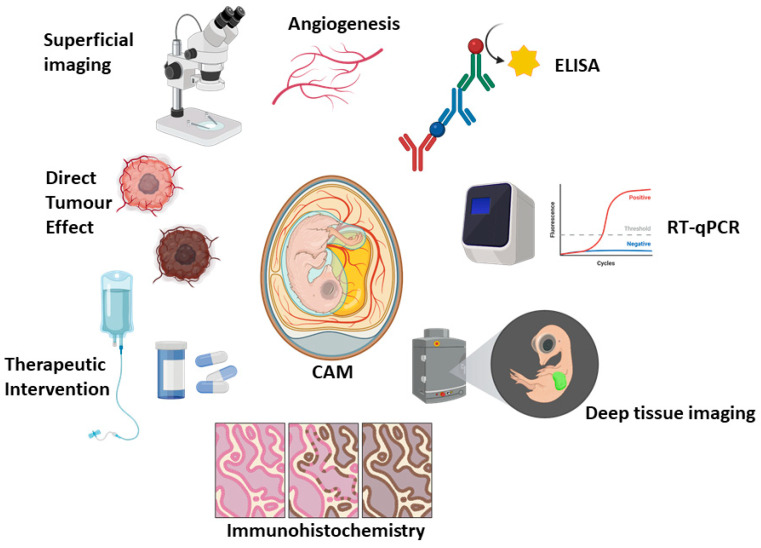
Representative outputs and utility of the CAM model. Created in BioRender. Cymer, I. (2026) https://BioRender.com/pj584g1, accessed on 27 November 2025.

Avian CAM xenograft models continue to redefine the experimental landscape in cancer research, standing as an agile and ethically compliant bridge between in vitro systems and mammalian in vivo models. Their ability to support tumour engraftment, vascularisation and therapeutic manipulation within a living organism positions them as a valuable complement to murine xenograft studies, offering a fast and cost-effective triage option to potentially reduce the number of mice used (rather than replace them completely). In the context of haematological malignancies, the CAM platform provides a unique opportunity to assess drug efficacy, toxicity, angiogenesis and tumour-microenvironment interactions in a dynamic, experimentally-accessible and cost-effective setting (Figure 1). Experimental considerations and variables between CAM versus mouse xenograft experiments are summarised in Table 4. Its simplicity, rapid turnaround, and compatibility with modern molecular and imaging tools make it a particularly attractive option for early-stage investigations and mechanistic exploration.

However, the CAM model remains under-utilised as a whole and therefore lacks standardisation, including (but not limited to) the field of haematological cancer research. Variations in experimental design, such as the day of implantation, can significantly affect tumour-developmental outcomes and model reproducibility, given the dependence on the cut-off point for embryo sacrifice. Similarly, methods of tumour cell delivery vary widely: some studies apply cells directly onto the CAM surface without a matrix; others use 3-D matrices to inoculate in situ, inject T-cells into the vasculature (a technically demanding and mortality-prone method), or transfer pre-formed “on-plants” generated in vitro. Additionally, others utilise only conditioned media to assess angiogenic responses, bypassing cell implantation entirely. While such methodological diversity reflects the adaptability of the model, it also complicates cross-study comparisons and obscures consensus on best practices. For example, no uniform agreement exists regarding the optimal implantation day—an especially important factor when injection techniques are employed, as late-stage manipulation of the allantoic vein has been associated with high embryonic death rates. This counteracts the model’s ethical advantages.

Another area requiring clarification concerns experimental replication. Across published studies, distinctions between biological and technical replicates are inconsistently defined. In the view of much of the CAM community, embryos derived from the same batch—identical in age, parental origin, and farm/hatchery—are considered technical replicates, not biological ones, given their shared developmental and genetic backgrounds. Establishing these distinctions will be essential for ensuring experimental transparency and reproducibility across laboratories.

Furthermore, although EU Directive 2010/63/EU provides clear ethical boundaries for CAM experimentation, national and institutional interpretations of these regulations vary, leading to inconsistencies in permissible experimental durations and endpoints across countries. Harmonising these ethical and procedural frameworks would enhance comparability.

In our view, progress towards standardisation would be best served by the establishment of a coordinated, multi-centre consortium in which harmonised experimental frameworks are prospectively tested across avian species and cancer types. Within such an approach, key variables, including egg strain and parental origin, inoculum preparation, optimal cell number, matrix composition, implantation site and defined implantation, manipulation and harvest days, could be systematically interrogated using shared standard operating procedures and predefined quality control metrics that the community agrees upon.

Furthermore, consensus is required that multiple tumours grown in one embryo should be treated as a set of technical replicates, with embryos produced under similar experimental conditions (same egg strain, age, parentage) being treated as additional technical replicates. Embryos from distinct parental lines are considered to be truly biologically replicated. Experimental rigour and integrity should be further preserved by ensuring that all tumours found in an embryo are subject to identical treatments, thereby avoiding pseudo-replication arising from mixed control and treatment conditions within the same biological system.

Additionally, regulation is required to provide consistency across countries in their interpretation of the cut-off values in the European Union Directive 2010/63/EU. Also, standardising the terminology used to distinguish between in situ CAM-grown tumours and angiogenesis assays using conditioned medium or pre-established “tumour” constructs, especially those established with pre-treatment strategies, will help to clarify ambiguities in concepts and enhance comparisons among studies.

Despite these challenges, the CAM model holds remarkable promise for advancing blood cancer research, as exemplified in this review. Its experimental flexibility positions it for high-throughput drug screening and mechanistic studies of tumour–stroma and tumour–vascular interactions. Furthermore, its compatibility with live imaging, molecular analysis and personalised therapy screening further strengthens its position as a translational bridge between cell culture and mammalian models. Importantly, by providing an in vivo context with less complexity and fewer regulatory constraints than higher-order mammalian systems, avian CAM models thereby enable rapid hypothesis testing, which can be easily integrated into prospective research—potentially accelerating discovery cycles in preclinical studies. As not all therapeutics can be effectively studied in vitro (e.g., therapeutic monoclonal antibodies, which require an active immune response to exert their effects), immunocompetent avian CAM models offer unique advantages over many immunodeficient mouse models traditionally used for xenograft studies. However, while mouse models may remain more suitable for long-term metastasis studies, strategic usage of CAM-based assays to pre-screen and optimise pharmacokinetic or toxicity parameters could have a positive impact on reducing the massive numbers of mice currently used for experimental cancer studies.

Looking forward, progress will depend on the establishment of shared standards to enhance model fidelity. As researchers continue to balance ethical refinement with experimental precision, the question may become not which model should prevail, but should the egg or the mouse come first? This rhetorical reflection encapsulates the evolving recognition that both systems are interdependent, each addressing distinct but complementary facets of preclinical discovery.

## Figures and Tables

**Table 4:** Comparison of CAM and mouse models for blood cancer research.

Parameter	Chick CAM Model.	Mouse Model (e.g., Xenograft, Syngeneic, GEMM, PDX)
Engraftment efficiency	High engraftment with efficiency dependent on implantation route and matrix support.	High engraftment rate for established cell lines in immunodeficient strains, with variable success for primary PDX (dependent on host strain and conditioning).
Time to detectable tumour/disease	Visible or imageable tumours and associated angiogenesis typically observed within 3–7 days post-engraftment.	Most murine models require 1–3 weeks for stable disease phenotypes, but this may extend to months.
Experimental turnaround	Completed within a single gestation cycle, yielding a total experimental duration of <18 days.	Weeks to months, accounting for engraftment, expansion, dissemination, treatment scheduling, and endpoint analysis.
Throughput and scalability	High throughput is enabled by low per-unit cost, minimal space requirements.	Throughput and scalability are limited by housing capacity, cost, and regulatory burden.
Cost per experimental unit	Low	High
Implantation modes relevant to blood cancers	Surface grafting enables mass formation and angiogenesis studies. Intravascular injection enables analysis of circulation, vascularization, etc.	Intravenous, subcutaneous, and intraosseous routes enable systemic dissemination, marrow tropism, organ-specific colonisation, etc.
Ability to model dissemination	Early dissemination can be detected in end organs.	Full dissemination cascades and long-term colonisation can be modelled over extended timeframes.
Bone marrow niche fidelity	Limited marrow-like microenvironment present in smaller avian models, restricting faithful modelling or marrow-dependent malignancies, unless niche-mimicking scaffolds are used.	High niche fidelity is achievable, humanised niches or stromal co-engraftment.
Immune context	Transient immune maturity.	Typically immunodeficient, though humanised models are available.
Imaging accessibility	Direct optical access, with high-resolution imaging of tumour-vascular interactions, supported with minimal instrumentation.	Deep imaging required with specialised modalities like bioluminescence imaging, magnetic resonance imaging, increasing technical and cost barriers.
Angiogenesis and vascular remodelling	Angiogenesis is a core strength of CAM models, with vessel patterning and remodelling directly visualised.	Angiogenesis studies are possible but require technically demanding intravital approaches.
Pharmacological relevance	Local delivery and rapid drug screens are straightforward, though technically demanding if intravenous routes are examined.	Systemic dosing, high metabolism, and toxicity modelling are applicable.
Best-fit applications	Rapid mechanistic studies, angiogenesis, early dissemination, direct tumour effect, and high-throughput drug screening.	Systemic disease biology, marrow dependence, immune therapies, resistance evolution.

## Data Availability

No new data were created or analyzed in this study.
